# Hyponatremia in traumatic brain injury patients: Syndrome of Inappropriate Antidiuretic Hormone (SIADH) versus Cerebral Salt Wasting Syndrome(CSWS)

**Published:** 2012-11

**Authors:** Parandoush Sepehri, Zahra Abbasi, Najaf Seid Mohammadi, Seyedreza Bagheri, Reza Fattahian

**Affiliations:** ^*a*^Department of Neurosurgery, Kermanshah University of Medical Sciences, Kermanshah, Iran.

## Abstract

**Background:**

Two common dysfunctions among traumatic brain injury (TBI) are hyponatremia secondary to the syndrome of inappropriate antidiuretic hormone secretion (SIADH) and cerebral salt wasting syndrome (CSWS).The present study was aimed to define real incidence and most common cause of this problem. Differentiation between these two syndromes is difficult because of overlapping signs, symptoms and specially laboratory data. Distinction between the two syndromes is based on patient's volume state. The present study aims to develop an alternative diagnosis strategy for defining the type of post-TBI hyponatremia.

**Methods:**

This was a single-center retrospective study conducted on TBI diagnosed patients referred to intensive care unit (ICU) of Taleghani Hospital (Kermanshah, Iran). Hyponatremia condition is diagnosed when sodium level reaches the values of less than 135 meq/lit. Basic criterion for diagnosing the hyponatremia type was only urine volume. Urine volume was compared with previous days and fluid intake. If the volume showed a reduction, then the patient was classified in SIADH group, and the prescribed treatment was only fluid intake restriction. In cases of CSWS that have polyuric state and hyponatremia, treatment is sodium and fluid replacement. CBC, Na, K, FBS, BUN, Cr and urine 24-hour volume were measured daily, while during the treatment, it was performed twice or more.

**Results:**

A total of 881 patients were referred to ICU during January 2011 to March 2012, of them, 678 patients had head trauma with and/or without other body injury. Out of all patients, 216 (%32) showed hyponatremia. Based on our diagnosis and treatment strategies, all of patient had lower urine output than previous day and were classified in the SIADH group and treated with only fluid restriction. None of patients were classified in CSWS group. All patients well recovered from hyponatremia with simple fluid restriction. In a clinical examination after a follow up period, the outcomes of all patients were acceptable with no adverse effects.

**Conclusions:**

Although CSWS was reported in many previous studies, the findings of the present study demonstrate that this procedure can be questioned and presence of CSWS following TBI is a rare condition, since it is probably an unpredictable response of kidney to stressor hormones.

**Keywords:**

Hyponatremia, SIADH, CSWS, TBI


					Volume 4, Suppl. 1 Nov 2012
Publisher: Kermanshah University of Medical Sciences
URL: http://www.jivresearch.org
Copyright:
Congress of Iranian Neurosurgeons, 14-16 November 2012, Kermanshah, Iran
Paper No. 17
Hyponatremia in traumatic brain injury patients: Syndrome
of Inappropriate Antidiuretic Hormone (SIADH) versus
Cerebral Salt Wasting Syndrome(CSWS)
Parandoush Sepehri a,*
, Zahra Abbasi a
, Najaf Seid-Mohammadi a
, Seyedreza Bagheri a
, Reza Fattahiana
a
Department of Neurosurgery, Kermanshah University of Medical Sciences, Kermanshah, Iran.
Abstract:
Background: Two common dysfunctions among traumatic brain injury (TBI) are hyponatremia secondary to the
syndrome of inappropriate antidiuretic hormone secretion (SIADH) and cerebral salt wasting syndrome (CSWS).The
present study was aimed to define real incidence and most common cause of this problem. Differentiation between
these two syndromes is difficult because of overlapping signs, symptoms and specially laboratory data. Distinction
between the two syndromes is based on patient's volume state. The present study aims to develop an alternative
diagnosis strategy for defining the type of post-TBI hyponatremia.
Methods: This was a single-center retrospective study conducted on TBI diagnosed patients referred to intensive care
unit (ICU) of Taleghani Hospital (Kermanshah, Iran). Hyponatremia condition is diagnosed when sodium level reaches
the values of less than 135 meq/lit. Basic criterion for diagnosing the hyponatremia type was only urine volume.
Urine volume was compared with previous days and fluid intake. If the volume showed a reduction, then the patient
was classified in SIADH group, and the prescribed treatment was only fluid intake restriction. In cases of CSWS that
have polyuric state and hyponatremia, treatment is sodium and fluid replacement. CBC, Na, K, FBS, BUN, Cr and
urine 24-hour volume were measured daily, while during the treatment, it was performed twice or more.
Results: A total of 881 patients were referred to ICU during January 2011 to March 2012, of them, 678 patients
had head trauma with and/or without other body injury. Out of all patients, 216 (%32) showed hyponatremia.
Based on our diagnosis and treatment strategies, all of patient had lower urine output than previous day and were
classified in the SIADH group and treated with only fluid restriction. None of patients were classified in CSWS group.
All patients well recovered from hyponatremia with simple fluid restriction. In a clinical examination after a follow up
period, the outcomes of all patients were acceptable with no adverse effects.
Conclusions: Although CSWS was reported in many previous studies, the findings of the present study demonstrate
that this procedure can be questioned and presence of CSWS following TBI is a rare condition, since it is probably
an unpredictable response of kidney to stressor hormones.
Keywords:
Hyponatremia, SIADH, CSWS, TBI
* Corresponding Author at:
Parandoush Sepehri: Department of Neurosurgery, Kermanshah University of Medical Sciences, Kermanshah, Iran. Tel: +989188316147
E-mail: ps_paris@yahoo.com, (Sepehri P.).

				

